# Supplementary material for: Study of Prescription-Indication of Outpatient Systemic Anti-Fungals in a Colombian Population. A Cross-Sectional Study
Source: Antibiotics (Basel). 2022 Dec 13;11(12):1805. doi: 10.3390/antibiotics11121805 (PMC9774786; doi:10.3390/antibiotics11121805)
Supplement: Supplementary file 1 [file antibiotics-11-01805-s001.zip › antibiotics-2019763-supplementary.pdf]

Supplementary Tables

Supplementary Table S1. Approved uses of systemic antifungals.

Antifungal	Approved use
**Flucytosine**	***Deep:*** Candidiasis (candidemia, endocarditis, urinary tract, pulmonary, endophthalmitis, central nervous system), cryptococcosis (meningeal, pulmonary)
**Fluconazole**	***Deep:*** Candidiasis (invasive, candidemia, central nervous system, esophageal, oropharyngeal, endophthalmitis, endocarditis, urinary tract, arthritis), cryptococcosis (meningeal, pulmonary), histoplasmosis (disseminated, central nervous system, pulmonary), coccidioidomycosis, blastomycosis, lymphocutaneous sporotrichosis , trichophytosis, cutaneous leishmaniasis<br>***Superficial:*** Vulvovaginal candidiasis, dermatophyte onychomycosis, pityriasis versicolor, ringworm
**Griseofulvin**	***Superficial:*** Vulvovaginal candidiasis, dermatophyte onychomycosis, ringworm
**Isavuconazole**	***Deep:*** invasive aspergillosis, invasive mucormycosis
**Itraconazole**	***Deep:*** Aspergillosis (bronchopulmonary, invasive, pulmonary), candidiasis (invasive, candidemia, esophageal, oropharyngeal), meningeal cryptococcosis, histoplasmosis (disseminated, central nervous system, pulmonary), coccidioidomycosis, paracoccidioidomycosis, blastomycosis, sporotrichosis, systemic chromomycosis.<br>***Superficial:*** Vulvovaginal candidiasis, dermatophyte onychomycosis, pityriasis versicolor, ringworm
**Ketoconazole**	Severe deep infections when other safer drugs are not available or are not tolerated
**Nystatin**	***Superficial:*** Candidiasis (vulvovaginal, cutaneous, mucocutaneous, oropharyngeal, gastrointestinal -not esophageal-)
**Posaconazole**	***Deep:*** Aspergillosis (bronchopulmonary, invasive, pulmonary), candidiasis (invasive, candidemia, esophageal, oropharyngeal).
**Terbinafine**	***Superficial:*** Dermatophyte onychomycosis, dermatophytosis (tinea corporis, cruris, pedis, capitis), pityriasis versicolor, seborrheic dermatitis, sporotrichosis (cutaneous, lymphocutaneous), cutaneous chromoblastomycosis
**Voriconazole**	***Deep:*** Aspergillosis (bronchopulmonary, invasive, pulmonary), candidiasis (invasive, candidemia, esophageal, oropharyngeal, endophthalmitis), blastomycosis, fusariosis, scedosporiosis.

Supplementary Table S2. Top 15 uses of systemic antifungals from 35,824 patients reporting primary and secondary diagnoses, Colombia.

Uses	Fluconazole		Nystatin		Ketoconazole		Terbinafine		Itraconazole		Fluconazole + secnidazole		Itraconazole + secnidazole		Voriconazole		Posaconazole		Isavuconazole	
	n=25340	%	n=5272	%	n=3949	%	n=796	%	n=588	%	n=102	%	n=48	%	n=38	%	n=18	%	n=4	%
Vaginitis - vulvitis – vulvovaginitis	12092	47.7	274	5.2	881	22.3	29	3.6	59	10.0	85	83.3	38	79.2	1	2.6	0	0.0	0	0.0
Opportunistic mycoses	2323	9.2	764	14.5	262	6.6	77	9.7	69	11.7	4	3.9	0	0.0	24	63.2	14	77.8	3	75.0
Onychomycosis	972	3.8	6	0.1	54	1.4	233	29.3	116	19.7	0	0.0	1	2.1	0	0.0	0	0.0	0	0.0
Pityriasis versicolor	707	2.8	16	0.3	435	11.0	38	4.8	60	10.2	0	0.0	0	0.0	0	0.0	0	0.0	0	0.0
Superficial mycoses	951	3.8	33	0.6	250	6.3	49	6.2	23	3.9	0	0.0	0	0.0	0	0.0	0	0.0	0	0.0
Acute nasopharyngitis	275	1.1	660	12.5	63	1.6	3	0.4	4	0.7	0	0.0	0	0.0	0	0.0	0	0.0	0	0.0
Unspecified mycosis	785	3.1	44	0.8	191	4.8	23	2.9	29	4.9	0	0.0	0	0.0	1	2.6	0	0.0	0	0.0
Body ringworm	776	3.1	6	0.1	208	5.3	49	6.2	25	4.3	0	0.0	0	0.0	0	0.0	0	0.0	0	0.0
Urinary infection	779	3.1	102	1.9	97	2.5	5	0.6	4	0.7	2	2.0	3	6.3	0	0.0	0	0.0	0	0.0
Acute tonsillitis	50	0.2	725	13.8	10	0.3	1	0.1	0	0.0	1	1.0	0	0.0	0	0.0	0	0.0	0	0.0
Stomatitis	46	0.2	742	14.1	8	0.2	0	0.0	0	0.0	0	0.0	0	0.0	0	0.0	0	0.0	0	0.0
Unspecified dermatophytosis	506	2.0	15	0.3	161	4.1	24	3.0	34	5.8	0	0.0	0	0.0	0	0.0	0	0.0	0	0.0
Bacterial or viral intestinal infections	184	0.7	208	3.9	20	0.5	3	0.4	1	0.2	0	0.0	0	0.0	0	0.0	0	0.0	0	0.0
Allergic/contact dermatitis	285	1.1	23	0.4	47	1.2	15	1.9	9	1.5	2	2.0	0	0.0	0	0.0	0	0.0	0	0.0
External otitis	252	1.0	27	0.5	63	1.6	1	0.1	4	0.7	0	0.0	0	0.0	0	0.0	0	0.0	0	0.0
